# You Don't Need an App—Conducting Mobile Smoking Research Using a Qualtrics-Based Approach

**DOI:** 10.3389/fdgth.2021.799468

**Published:** 2022-01-06

**Authors:** Yong Cui, Jason D. Robinson, Rudel E. Rymer, Jennifer A. Minnix, Paul M. Cinciripini

**Affiliations:** Department of Behavioral Science, The University of Texas MD Anderson Cancer Center, Houston, TX, United States

**Keywords:** mobile intervention, smoking research, qualtrics, mHealth, digital health, smartphone app, ecological momentary assessment

## Abstract

With the increasing availability of smartphones, many tobacco researchers are exploring smartphone-delivered mobile smoking interventions as a disseminable means of treatment. Most effort has been focused on the development of smartphone applications (apps) to conduct mobile smoking research to implement and validate these interventions. However, developing project-specific smartphone apps that work across multiple mobile platforms (e.g., iOS and Android) can be costly and time-consuming. Here, using a hypothetical study, we present an alternate approach to demonstrate how mobile smoking cessation and outcome evaluation can be conducted without the need of a dedicated app. Our approach uses the Qualtrics platform, a popular online survey host that is used under license by many academic institutions. This platform allows researchers to conduct device-agnostic screening, consenting, and administration of questionnaires through Qualtrics's native survey engine. Researchers can also collect ecological momentary assessment data using text messaging prompts with the incorporation of Amazon Web Services' Pinpoint. Besides these assessment capabilities, Qualtrics has the potential for delivering personalized behavioral interventions through the use of JavaScript code. By customizing the question's web elements in Qualtrics (e.g., using texts, images, videos, and buttons), researchers can integrate interactive web-based interventions and complicated behavioral and cognitive tasks into the survey. In conclusion, this Qualtrics-based methodology represents a novel and cost-effective approach for conducting mobile smoking cessation and assessment research.

## Introduction

Smoking is a well-established risk factor for various types of cancer, diabetes, and cardiovascular diseases and accounts for 480,000 deaths each year in the United States, making it the leading cause of preventable death (1). In addition to health-related adversities, smoking imposes an enormous cost of over $300 billion in healthcare spending and lost productivity (2). Despite considerable reductions in use over the past several decades, smoking prevalence in the United States is still high: 15.6% of men and 12.0% of women are current cigarette smokers (3). Furthermore, the prevalence of smoking is more concentrated among vulnerable populations, such as those with mental health disorders (4) and low socioeconomic status (5). Reducing smoking's prevalence and its societal impact requires the development and implementation of smoking cessation interventions at the population level.

## mHEALTH in Smoking Research

One approach with a potential population-wide reach is mobile health (mHealth)-based smoking intervention. Conventional mobile interventions have been implemented in the form of text messages [i.e., short message service (SMS)], and accumulating evidence suggests that SMS-based cessation programs are effective in promoting abstinence among smokers (6). However, with the advancement of mobile technology, penetration of smartphones has continually increased, with a current ownership rate of over 80% among all Americans (7), 76% among people with <$30,000 household income (7), and 60% among people with serious mental illness (8). The increasing availability of smartphones, particularly among those vulnerable populations in which smoking is more concentrated, makes the smartphone technology a viable choice for providing evidence-based treatments. Compared to text messages, smartphones, through various applications (apps), provide more engaging content and assessment capabilities, and thus they have become an attractive platform for developing novel mobile smoking cessation interventions.

Consistent with the smartphone trend, several 100 smoking cessation-related apps have been introduced, but most of these apps lack valid theoretical underpinning or clinical data to support their use and efficacy (9–12). To address this shortcoming, several tobacco researchers have developed evidence-based smartphone apps for smoking interventions. For example, Bricker et al. developed a smartphone app that delivers acceptance and commitment therapy for smoking cessation, and they obtained clinical data showing its efficacy (13, 14). A group led by Businelle evaluated an ecological momentary assessment (EMA)-based just-in-time adaptive intervention (15, 16), and they have successfully identified key predictors of smoking lapses (17).

In smoking research, researchers also administer behavioral tasks to study the underlying neurocognitive processes associated with smoking and its intervention. For instance, researchers have found that smokers show attentional bias toward smoking-related cues using several behavioral paradigms, including the dot-probe (18, 19), Stroop (20, 21), and rapid visual information processing (22) tasks. Other researchers have extended these laboratory findings and evaluated their potential for treating smoking using mHealth platforms. Recently, our group has studied the effect of attentional bias modification training on smoking behaviors and cessation outcomes and found that 2-week smartphone-delivered attentional bias modification training led to reduced attentional bias to smoking cues but did not have any effect on abstinence (23).

## The Rationale

In general, developing smoking cessation apps or behavioral tasks not only requires sophisticated programming skills and thus more development time, which may delay the launch of the study, but may also involve high development costs, placing additional constraints on conducting mHealth tobacco research. Moreover, in conducting mobile smoking research, investigators often administer various questionnaires to study changes in smoking-related phenotypes, such as nicotine withdrawal and craving. Implementing assessments tailored to a study's specific research questions can further complicate the app development process.

To address the difficulty of developing study-specific smartphone apps, particularly when resources are constrained (e.g., a pilot study without substantial funding), we describe an approach for carrying out mHealth tobacco research by taking advantage of the Qualtrics (24) survey platform (Qualtrics LLC, Provo, Utah, USA). Unlike the smartphone apps that need to be tailored to different mobile platforms, Qualtrics is platform-agnostic because it runs within the smartphone's built-in web browser app. More importantly, Qualtrics service is used under license by many research institutions (25), meaning that researchers can often leverage it as an institutional resource to conduct survey-based research with little or no extra expenses.

Beyond supporting conventional question-based survey research, Qualtrics offers advanced features, such as unique survey links, programmatic response notification, and downloading of the response data, and these features can be used to facilitate several aspects of mHealth tobacco research, such as EMA. While Qualtrics itself can be used for many processes involved in mHealth tobacco research, it requires additional entities, including Amazon Web Services (AWS; Amazon.com, Seattle, Washington, USA) and programming skills (e.g., JavaScript), to take full advantage of its capabilities and related scalability.

## The Present Report

Over the past several years, we have been conducting multiple smoking studies, including NCT02964182 and NCT04604509 (clinicaltrials.gov registrations), in which we have used Qualtrics as the core data collection tool. These studies were approved by the institutional review board (IRB) at The University of Texas MD Anderson Cancer Center. Each study has used some, but not all, elements of the approach presented here.

Thus, to fully describe this methodological approach independent of specific research questions, here we focus on a generic hypothetical study that is similar to our ongoing smoking studies, but with a simpler design. Please note that the present hypothetical study was never implemented. Our intention is to frame this hypothetical study with the essential and generalizable research elements as the skeleton, thereby allowing us to provide a high-level technical guidance for the implementation of the Qualtrics-based approach.

We recognize that there are other survey or data-collection engines, such as REDCap (26), and the present report is not intended to provide a direct comparison between Qualtrics and these alternatives. Instead, we share our experience of using the Qualtrics-based data collection tools after trial and error through multiple smoking studies, in the hope of facilitating mobile smoking research. More generally speaking, this approach will also benefit behavioral researchers who have Qualtrics access in other domains besides tobacco.

## The Hypothetical Study

### Overview

Suppose the goal of a hypothetical study was to investigate smokers' affective fluctuations over the medication-aided cessation process, and that EMA were included to collect affect-related data. Further, suppose that the intervention used in the hypothetical study included nicotine replacement therapy (NRT) medication (i.e., nicotine patches), phone-based behavioral counseling, and real-time relapse prevention (see section Ecological Momentary Inventions for more details). The inclusion of self-reported questionnaires and the EMA-based relapse prevention element allow us to show the full capability of this approach for supporting mobile smoking research using Qualtrics both for assessment and intervention delivery.

To provide a coherent reading flow, we used the past tense consistently in this report. To demonstrate the strength of using Qualtrics for remote assessment, this hypothetical study was described as an all-digital trial with all staff-participant interactions conducted remotely. The study advertisements were run throughout the state, which allowed us to collect a large representative sample to leverage the strength of this mHealth platform for high levels of recruitment.

### Visit Structure

The study consisted of five visits (Table 1). First, interested participants completed an initial web screening to provide their basic demographic and smoking information. Second, if potential participants met the initial eligibility criteria, our staff conducted a phone interview to determine their final eligibility. If eligible, participants were enrolled into the study after completing the consent process. A staff member then sent the nicotine patches to the participant by mail. Participants started the NRT at Visit 3. The NRT intervention lasted 4 weeks, ending at Visit 4. Between Visit 3 and Visit 4, participants completed their EMAs, which included a daily diary assessment, random assessments, and participant-initiated assessments. One month after the treatment, participants completed Visit 5 by phone as their end-of-study visit.

**Table 1:** The visit structure for the hypothetical study.

**Visit**	**Days**	**Key events**
1	-	Initial web screening
2	0	Phone interview, consent, enrollment, baseline IAT
3	7	Start NRT, phone-based counseling, quit smoking, start EMA
4	35	EOT, end EMA, phone-based counseling
5	63	1-month follow-up, end of study

*NRT, nicotine replacement therapy; IAT, implicit association test; EMA, ecological momentary assessment; EOT, end of treatment*.

## Qualtrics Surveys

### Screening Survey

The screening survey collected the basic demographic (e.g., name, contact information, age, and race/ethnicity) and smoking-related (e.g., smoker status and daily cigarette consumption) information. By design, this Qualtrics survey was generic so that all our smoking-related studies could use this survey and benefit from a single shared recruitment campaign effort. All the information was used to determine participants' initial eligibility. When found potentially eligible, our staff arranged a follow-up telephone interview to evaluate their full eligibility.

### Consent Survey

The Qualtrics consent survey consisted of two parts: (1) a detailed description of the present study and (2) the IRB-approved consent statement. At the end of the consent statement, participants could give their consent by providing their electronic signature (Qualtrics supports signature as a question type).

### Visit-Specific Survey

For each of the Visits 2 to 5, we used SMS to send a visit-specific survey to each participant for them to complete on Qualtrics. For simplicity, the survey consisted of only the Questionnaire of Smoking Urges-Brief (QSU-Brief) (27) and the Positive and Negative Affect Schedule (PANAS) (28). In addition to these two questionnaires, the survey for Visit 2 included the smoking-related implicit association test (IAT), which is a behavioral paradigm to measure the implicit attitudes toward smoking-related cues (29, 30). The inclusion of this behavioral assessment in our hypothetical study allows us to demonstrate how to integrate behavioral tasks into the present Qualtrics-based approach.

In the smoking IAT, participants were asked to classify words in the categories of pleasant and unpleasant and images in the categories of smoking-related and neutral using two opposite classification labels, which varied between the seven blocks (Table 2). We measured participants' reaction time in milliseconds of classifying the stimuli to measure whether participants had faster classification when smoking was associated with the unpleasant category than when smoking was associated with the pleasant category. For more information on the IAT methodology, please refer to the pertinent reviews (31, 32).

**Table 2:** A representative smoking IAT experiment.

**Block**	**Trials**	**Classification labels (left vs. right)**
1	20	Pleasant vs. unpleasant
2	20	Smoking vs. non-smoking
3	20	Pleasant or smoking vs. unpleasant or non-smoking
4	40	Pleasant or smoking vs. unpleasant or non-smoking
5	20	Non-smoking vs. smoking
6	20	Pleasant or non-smoking vs. unpleasant or smoking
7	40	Pleasant or non-smoking vs. unpleasant or smoking

*In an IAT trial, a word or a picture is shown at the center of the screen. The participant is asked to press a button to classify the word or the picture according to the two labels shown on the screen. The response and the reaction time are recorded. IAT, implicit association test*.

### EMA Surveys

#### Daily EMA Diary Survey

Between Visit 3 and Visit 4, participants completed a daily diary EMA on Qualtrics. The diary survey assessed their daily cigarette, alcohol consumption, and nicotine patch usage for the previous day, and their affective states using PANAS.

#### Random EMA Survey

During the EMA period, participants were prompted by the SMS to complete a random assessment each day. The random survey included the following measurements: (1) the time of their most recent cigarette if it was within the last 24-h window (i.e., Did you smoke any cigarettes in the last 24 h? If the answer was yes, the following question was displayed: What time did you smoke your last cigarette in the last 24 h?), (2) the time of their most recent patch usage (i.e., When did you use your most recent nicotine patch?), and (3) their current craving levels using a five-point Likert scale (i.e., How much are you craving for a cigarette now? Choices: 1. Not at all, 2. A little, 3. Moderate, 4. Very much, 5. Extreme).

#### Participant-Initiated EMA Survey

During the EMA period, participants had access to the participant-initiated (i.e., spontaneous) EMA survey. In this survey, participants could record a smoking event or a craving episode and the primary trigger (e.g., others smoking and anxiety). If it was a craving episode, participants could also enter the severity of the craving using a five-point Likert scale as used in the random EMA survey.

Per protocol, participants were instructed to report craving episodes as often as they needed to. As a relapse prevention strategy, when participants reported any level of craving other than not at all, the survey displayed a text message or a short video clip that was designed to prevent smoking lapses. The contents of the text messages and video clips involved a range of topics, such as benefits of quitting, suggestions of alternative things to do, motivational testimonials, and stress coping. The content of the text message or video clip was determined by the trigger that the participant had picked.

## The Building Components of the Approach

### The Qualtrics Platform

Through our institute's group license, Qualtrics was used to create and distribute the surveys. Qualtrics supports all the common question types in a typical research survey, including multiple choice, text entry, slider (to select a value in a continuous range), rank order, and matrix table (multiple items with shared multiple choices). Besides these standard questions, Qualtrics supports applying JavaScript code to customize questions by configuring additional HyperText Markup Language (HTML) elements that are not natively present in the standard questions, meaning that we can run more complex behavioral tasks, such as the IAT used in the present hypothetical study.

Qualtrics allows us to create unique survey links for each of the participants' surveys. When participants complete the survey, all the responses are immediately saved to Qualtrics's server, from which we can download the response data as a text file for further offline data processing and analysis as well as assessment of compliance.

Notably, Qualtrics provides several application programming interfaces (APIs), and we, as well as other researchers with coding skills, can interact with the Qualtrics server programmatically. For instance, instead of using the Qualtrics website to download a survey's responses, one can download these responses using the appropriate API.

### AWS Pinpoint

AWS offers a wide range of cloud computing-related services, among which Pinpoint is relevant to the present approach. AWS Pinpoint is designed to handle two-way communications in a variety of forms, such as email, SMS, and push notifications. For the present hypothetical study, we used AWS Pinpoint to handle outbound and inbound SMS. One essential purpose of the outbound SMS was reminding participants to complete the surveys, including visit-specific ones and EMA-related surveys.

To facilitate scalability, we used Pinpoint's API to schedule and process SMS messages programmatically. However, please note that Pinpoint offers web interfaces to users without programming expertise to handle SMS in a manual manner.

### The Microsoft SQL Server

We use a HIPPA-compliant institutionally managed Microsoft SQL server for our research. The server has two key components: the database and web services. The database holds the data tables for the study's data, while the web services are responsible for handling web-related traffic between our database and external entities, including Qualtrics and AWS.

Depending on the specific design of a study, the database can have many data tables other than the ones shown below. For illustrative purposes, here we describe the key data tables for the hypothetical study (Table 3).

**Table 3:** Data tables in the SQL database.

**Table name**	**Description**
Subject	The basic information about study participants, including subject ID, mobile phone number, and email
Visit	The visit dates for each subject
QSU	The QSU-Brief scores by subject and visit
PANAS	The PANAS scores by subject and visit
IAT	The IAT data saved at the block level
Abstinence	The point-prevalence abstinence data for multiple time points
Daily_EMA	The data for the diary EMA, including daily cigarette, patch use, and affective measures
Random_EMA	The data for the random EMA, including the current craving level, recent smoking event, and patch usage
Spontaneous_EMA	The data for the spontaneous EMA, including the nature of the event (smoking vs. craving) and the trigger
Survey	The deployed Qualtrics surveys
Distribution	The unique survey links by subject. This table will also track the response ID and the status.

*QSU, questionnaire of smoking urges; PANAS, positive and negative affect schedule; IAT, implicit association test; EMA, ecological momentary assessment*.

As a side note, although we use the Microsoft SQL Server hosted by our institution, researchers who wish to implement the present approach can consider using AWS as their database host if they cannot deploy their own server due to resource limitations. Equally important to note is that besides AWS, other major cloud computing companies, such as Microsoft and Google, also provide scalable on-demand database hosting services.

### Web Service APIs

We built two sets of web service APIs. The first set is responsible for communications between our database and the Qualtrics, and the other set is for communications between our database and the AWS. For subsequent referencing, we include the API names as part of the titles.

#### Generate Unique Survey Links (GenerateSurveyLinks API)

This API invokes several pertinent Qualtrics APIs to generate unique survey links, allowing us to track the completion status for each survey sent to the participants. We also set a proper expiration date for these links such that we could prevent participants from completing a survey outside of the allowed time window. Using this API, we generated unique links with each link associated with a unique distribution ID for all the surveys, except for the initial web screening survey, which served as a public data entry point to screen all potential participants and thus whose link needed to be static for consistent public access. The generated unique survey links were saved to the Distribution table.

#### Handle the Notification of a Completed Response (NotifySurveyCompletion API)

**Figure 1:**
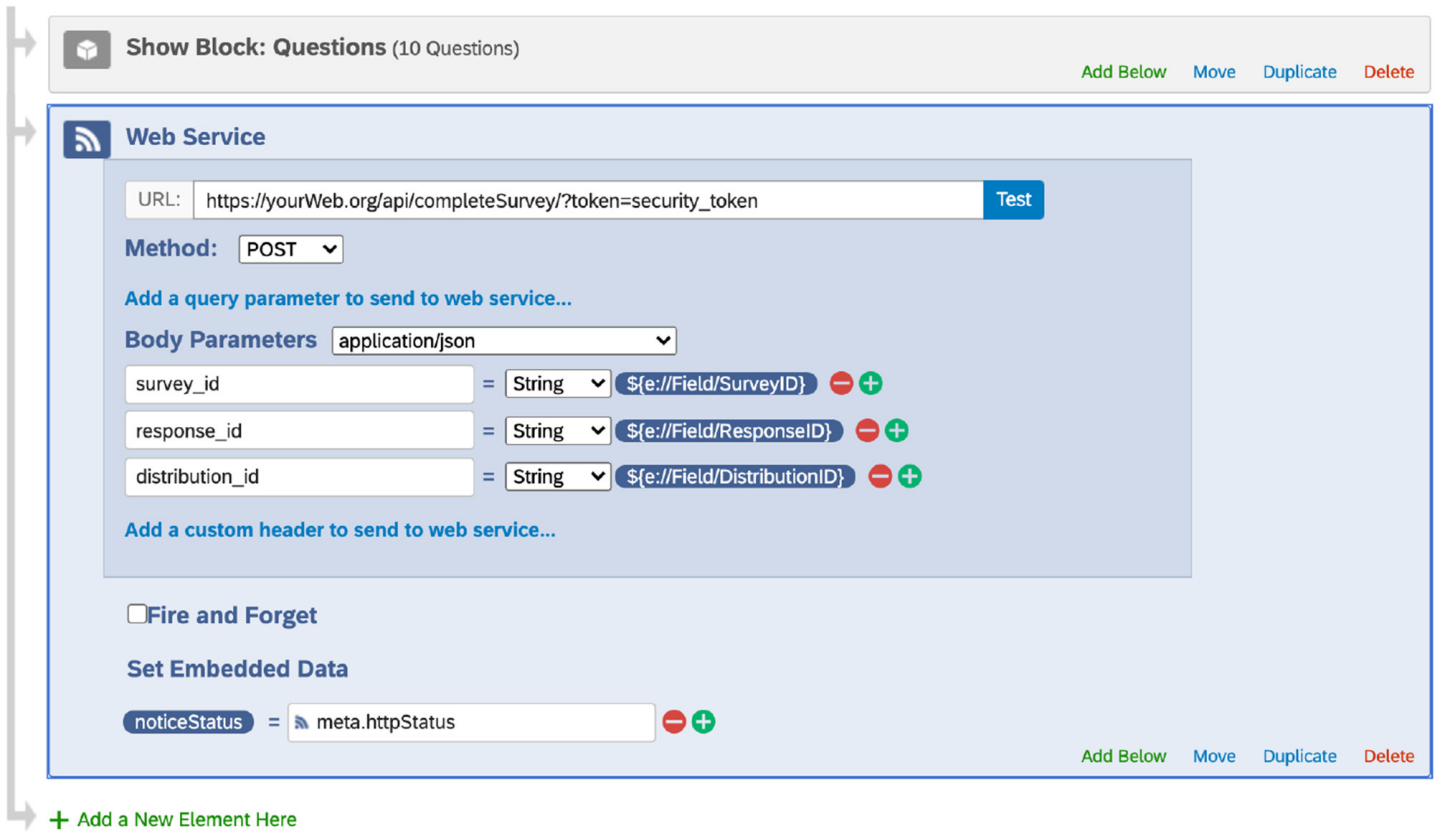
Adding the web service element at the end of a survey (screenshot taken from the Qualtrics website under The University of Texas MD Anderson Cancer Center's institutional Qualtrics license). This web service request sends the survey ID, response ID, and distribution ID to the database, which is used to update the Distribution table. Note that Qualtrics may update its interface.

At the end of each Qualtrics survey, we added a flow control Web Services element (Figure 1). Specifically, this element submitted a POST request to send the survey ID, response ID, and distribution ID to our server. Our web services handled this information by updating the Distribution table. When the update succeeded, the web services returned a success code, and Qualtrics saved the code as an embedded data field in the final response data file. When the update failed, a failure code was returned and saved instead.

With this API, our database was kept synchronized with all the completed responses. More importantly, by cross-referencing the scheduled surveys, we could track the completion status of each survey and generate reports on the survey completion rates.

#### Download Response (DownloadSurveyResponse API)

Once a survey was completed, our server used the saved survey ID and response ID to download the corresponding response by calling pertinent Qualtrics APIs. The response included all the embedded data, particularly the unique distribution ID. This distribution ID was used to determine the type of the survey and the responder (i.e., the participant) such that we could process the response and save the data to the proper destination tables (e.g., PANAS) with the data associated with the right participant.

Before we implemented the notifySurveyCompletion and downloadSurveyResponse APIs, we had a different API that downloaded responses every night, preventing us from tracking survey response status in real-time. Later, we realized that it could be a limiting factor if we would carry out any research that was dependent on the response completion. Thus, we built these two APIs, which allowed us to achieve real-time data synchronization between completed responses and scheduled responses in the database. From the perspective of intervention delivery, tracking a participant's response completion status and the detailed response results provides the basis for developing tailored real-time intervention.

#### Send Outbound SMS (SendMessage API)

To send an SMS to a participant, our server used the AWS Pinpoint API by specifying the recipient's mobile phone number and the message content. Another important parameter was the scheduled time for the SMS. AWS Pinpoint can send the SMS at a specified time, and this feature allowed us to schedule a future SMS.

#### Process Inbound SMS (ProcessMessage API)

When a participant sent us an SMS, the SMS together with the sender's mobile phone number was routed to our server through AWS Pinpoint. Our server ran a database query to identify the participant who sent the message using the mobile phone number. The information, including subject ID, phone number, and message content, was forwarded to the study staff *via* an email so that they could handle the question/request accordingly.

Once the notification was sent to the study team by our server, it initiated an outbound SMS to inform the participant that we had received his/her message and that a staff member would respond to them within one business day. The participant was also instructed to contact the study team by phone for any urgent issues or call 911 directly if it was a medical emergency.

## Implementation of the Research Components

### Recruitment and Screening

**Figure 2:**
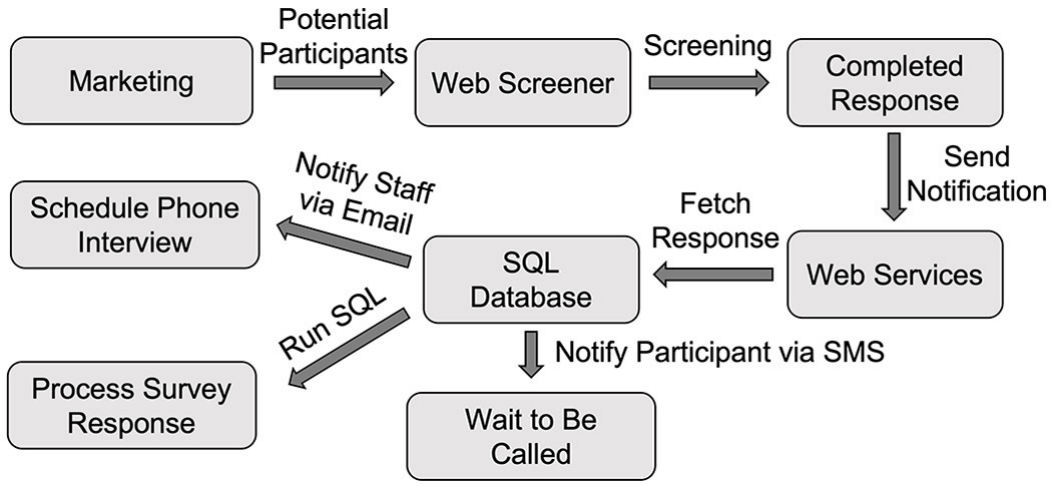
The Workflow of initial screening using Qualtrics. SQL, Structured Query Language.

The web screening survey's link was placed on our study recruitment web page. By clicking the link, potential community participants were directed to the web screening survey page. Once they completed the survey, Qualtrics invoked our web services API (notifySurveyCompletion) to notify us of the completed response, and our server could then fetch the response data from the Qualtrics server using the downloadSurveyResponse API. Three operations were performed once the response data were downloaded (Figure 2).

First, our server processed the response and saved the data into the Subject table. Second, our server sent an email to the study staff to notify them of the newly completed response so that they could schedule a phone interview to conduct an additional interview. Third, a text message to the potential participant was sent to keep him/her informed about the upcoming phone interview call using the sendMessage API through AWS Pinpoint.

### Identification and Consent

The identification and consent procedure can vary between institutes per their respective IRB requirements. Researchers need to consult with their IRB regarding the approved procedures. Here, from a technical perspective, we describe a possible implementation for obtaining identification and consent and we also provide alternative procedures where applicable.

To identify a participant, we used the IRB-approved videoconferencing software, such as Zoom (Zoom Video Communications, Inc., San Jose, California, USA), to conduct an onboarding call. When our staff scheduled the call, they created a Zoom meeting event and shared the event (e.g., the meeting's time and link) with the participant *via* email or SMS. The participant could have the call with the staff using either the Zoom app if they installed it or the phone's built-in web app. During the Zoom call, our staff asked to see a proper identification document to verify the participant's identity. The staff manually recorded a screenshot of the identification document for record-keeping purposes. Alternatively, we could also ask the participant to upload a photocopy of the identification document. Because Qualtrics supports file uploading as a question type, we embedded the identification request as part of the screening survey.

To obtain the consent, we sent the participant the consent survey that contained the required consent statement. In the survey, we requested the participant's signature as a question. If necessary, our staff had a telephone or videoconferencing call with the participant should any questions arise while the participant was completing the survey. Alternatively, if the IRB requires formal electronic signatures in a document, researchers can also investigate DocuSign (DocuSign, Inc., San Francisco, California, USA), which has established APIs to streamline the collection of multiple signatures in a single document.

### EMA

In this study, participants were asked to complete a daily diary, one random assessment, and participant-initiated spontaneous assessments during their EMA period between Visits 3 and 4. For these three kinds of assessments, we used three surveys, namely diary, random, and participant-initiated, as described in the surveys section.

### Daily Diary EMA

**Figure 3:**
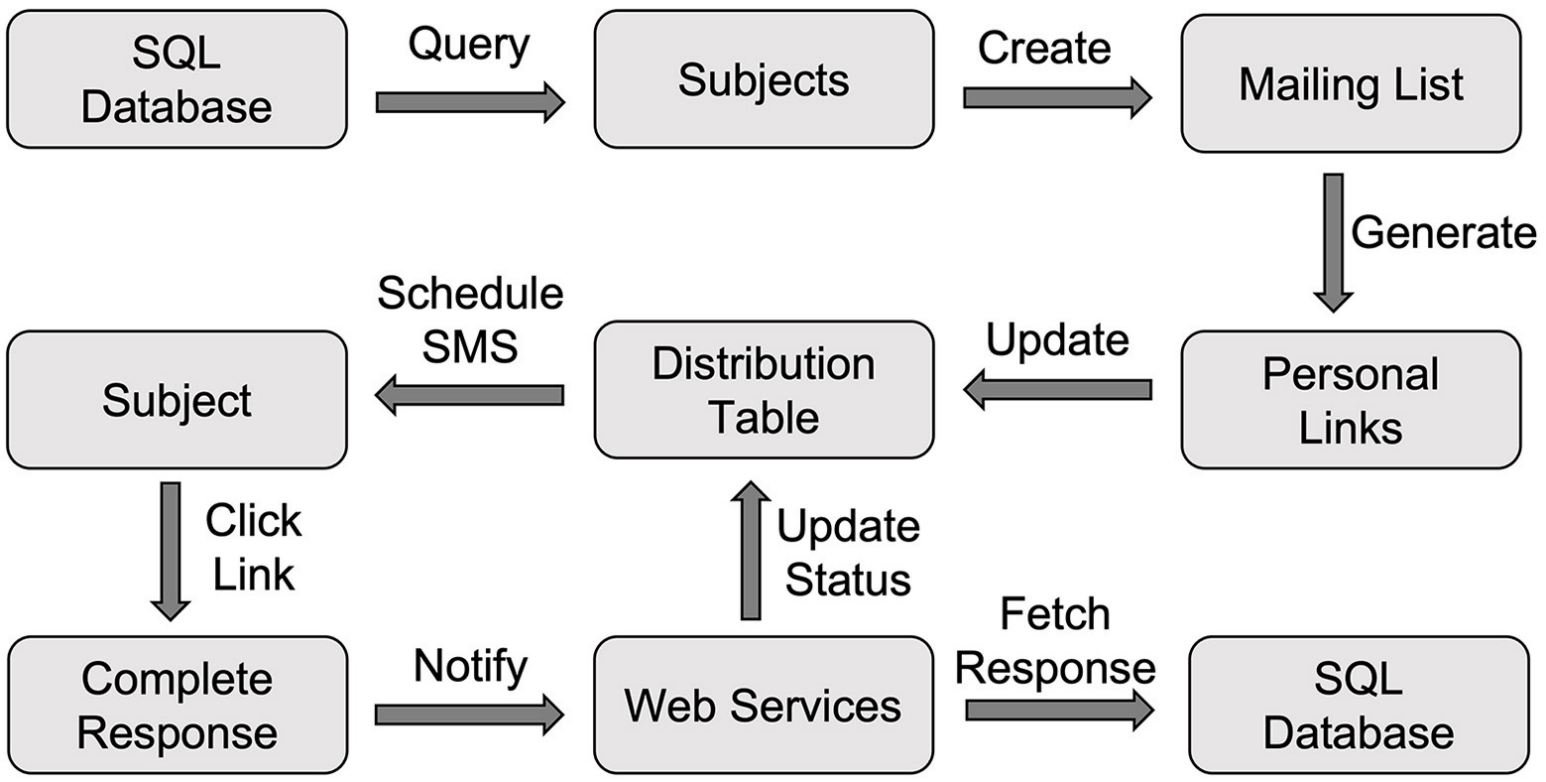
The Workflow of conducting EMA using Qualtrics. EMA, Ecological Momentary Assessment.

For the daily assessment, in the early morning (e.g., 2 a.m.) each day, our database was scheduled to run a query to generate the list of participants who were scheduled to have their EMA on that day. We used the generateSurveyLinks API to create a distribution list, which included unique survey links for each participant (Figure 3). To prevent participants from using an old link that was outside the time window, we set the proper expiration dates to these links, after which these links became inactive. For instance, the daily assessment survey link expired at midnight that evening.

These unique links were saved to the Distribution table in our database. At the set time in the morning (e.g., 9 a.m.), we used our sendMessage API to schedule an SMS through AWS Pinpoint to each participant, and the message contained the unique survey link and reminded them of completing the daily diary. Once the participant completed the diary survey, the response was captured by Qualtrics. As shown in Figure 1, the web service element at the end of the Qualtrics survey invoked the notifySurveyCompletion API to update the status of this distribution and recorded the completion time in the Distribution table. The response was also downloaded using the downloadSurveyResponse API and processed to save the data to the Daily_EMA table.

To personalize the daily assessment time based on the participant's routine, researchers can optionally record the preferred daily assessment time for each participant and save it in the Subject table. This preferred timepoint would then be saved as the scheduled time field in the Distribution table, and the SMS for reminding daily EMA was scheduled to be sent at that time.

When setting up the SMS reminders for EMA, we encountered an issue in which the generated personalized EMA survey links were too long to fit into a single SMS message (i.e., they exceeded the 160-character limit). Although having multiple SMS as a reminder is probably not a concern for researchers implementing SMS as EMA prompts because most modern phones can handle message concatenation to combine multiple SMS messages into a single one, an SMS with an excessively long link could still confuse participants. Thus, we developed another line of functionality that shortened these long links, such that we could send the links together with other necessary content (e.g., instructions to “complete your daily diary”) within a single SMS. Researchers who wish to implement the link shortening feature can look into AWS's CloudFront, a cloud computing service provided by AWS for handling link shortening, re-directing, and other web link interaction-related services.

### Random EMA

The procedure for conducting the random assessment was similar to that for the daily assessment, with only one difference. Instead of scheduling the SMS at a fixed timepoint (or the one specific to the participant), our database generated a random timepoint for each participant, and this timepoint was used to schedule the SMS through AWS Pinpoint, which sent out the message at the specified time. The timepoint was saved to the scheduled time field in the Distribution table.

### Participant-Initiated EMA

Unlike daily and random assessments, the timepoints of which were determined by the study, spontaneous assessments were initiated by the participant. Instead of using SMS reminders, we created a unique link to the survey for each participant to allow multiple responses. This way, the participant could initiate the spontaneous survey as many times as they wanted without invalidating the link upon its completion.

To facilitate participants' access to the survey, the staff instructed them to create a shortcut to the survey using the provided link on their phones' home screen. Placing a website link shortcut on the home screen is supported by major mobile operation systems, including Android and iOS. As an important note, similar to the daily and random assessments, the spontaneous assessment's link contained embedded data, including the distribution ID, which allowed us to know which response went with which participant.

### Ecological Momentary Interventions

**Figure 4:**
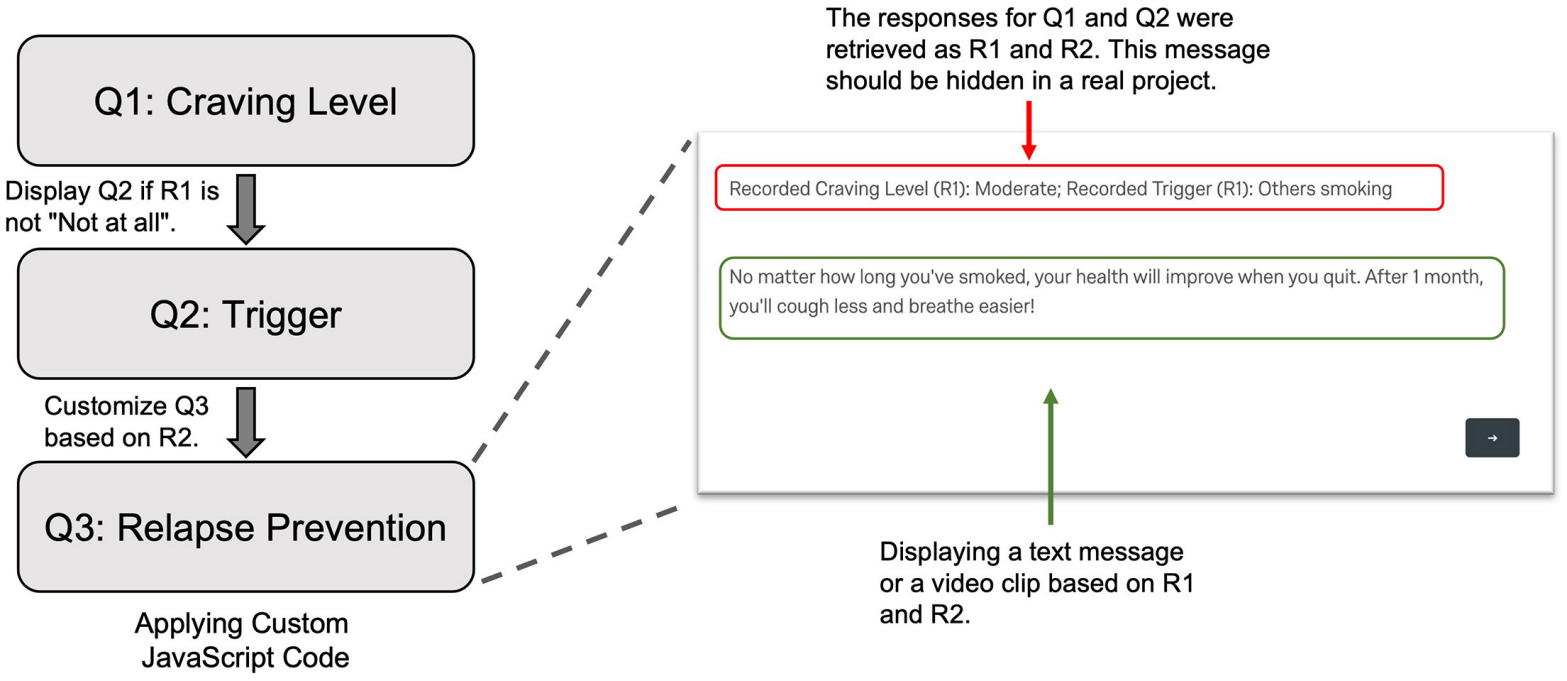
Display a text message or video clip as a relapse prevention strategy in the subject-initiated EMA. Q1 was the question assessing current craving level. When the subject reported a carving level other than “Not at all,” Q2 was displayed to access the primary trigger. In Q3, using custom JavaScript code, we retrieved the responses to Q1 and Q2 as R1 and R2, respectively. Based on these values, we selected a proper text message or a video clip to show to the subject. EMA, Ecological Momentary Assessment.

As an example of providing ecological momentary interventions (EMI), we included a relapse prevention component that was built upon participant-initiated EMA. Specifically, to prevent relapses, when participants reported any craving, the participant-initiated EMA displayed a text message or video clip to help them resist craving. To do that, we applied custom JavaScript to a Text/Graphic question (Q3 in Figure 4). In the code, we retrieved the participant's responses to the questions about the craving level and primary trigger for the craving episode. Based on the trigger, we displayed a text message or video clip that specifically addressed the trigger.

### Behavioral Tasks

**Figure 5:**
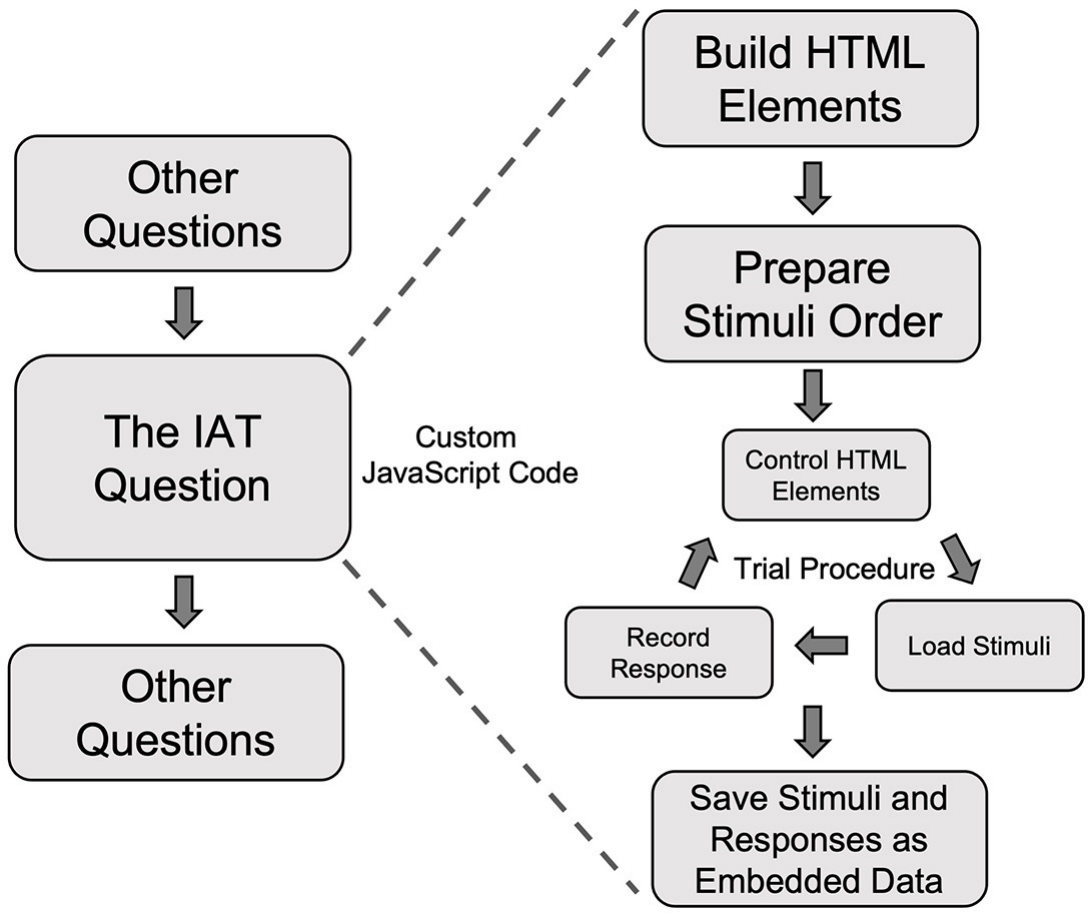
A schematic flow of using custom JavaScript code to create the Implicit Association Test in a Qualtrics survey. IAT, Implicit Association Test.

In the present study, participants completed the IAT as part of the visit-specific survey for Visit 2. To integrate the IAT into the survey, we added JavaScript code to customize a question to control the presentations of the stimuli and record participants' responses (Figure 5). Building a behavioral task, such as the IAT, as a question in Qualtrics involved the following general steps.

### Build the HTML Elements

**Figure 6:**
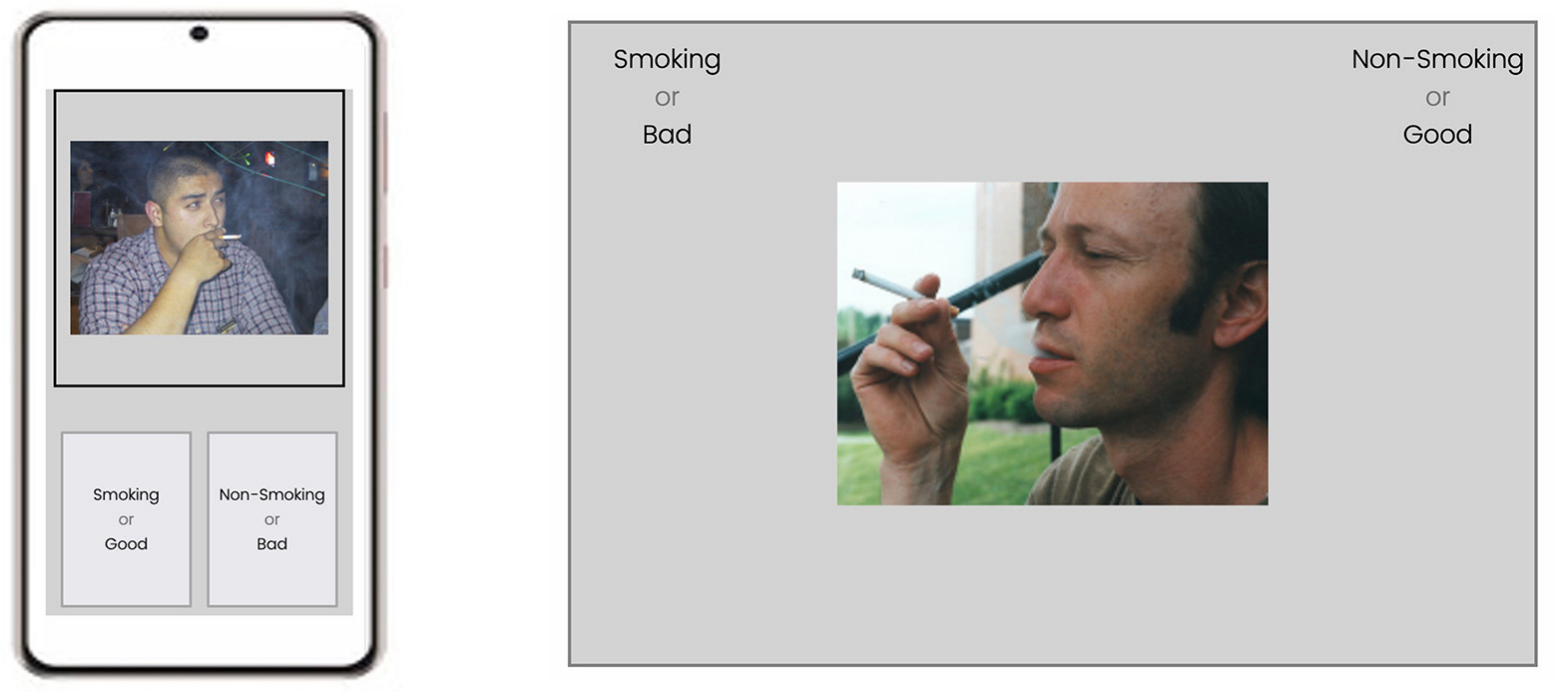
A schematic representation of a trial in the implicit association test experiment running a smartphone **(left)** and on a desktop computer **(right)**. IAT, Implicit Association Test.

By default, Qualtrics's built-in question does not have the HTML elements that we need to run a behavioral task. Thus, the first step is to add the necessary HTML elements to the web page. In the case of the IAT experiment, the key HTML elements included two boxes to show the classification labels at the bottom of the smartphone's screen and one stimulus presentation box to show images or words at the top of the screen (Figure 6, left). Please note that if the researchers wish to run the IAT task or any other behavioral task, they need to configure the interface separately. As an example, the interface running on a computer should be specifically configured to utilize a computer's larger screen (Figure 6, right) compared to a smartphone's.

We enabled the recording of the keyboard or touch events (for smartphone devices), such that we could record participants' responses to determine whether the response was correct. Another key data point we recorded was the reaction time for each trial, which was the time between the onset of the stimulus and the response event.

### Prepare Stimuli

The classic smoking IAT experiment consists of seven blocks in which participants classify smoking and neutral images and/or pleasant and unpleasant words based on the classification labels. We determined the order of the blocks and stimuli following the pre-set rules (e.g., randomization and counterbalancing) of running an IAT experiment. The pre-determined order allowed the program to proceed by directly retrieving information for the next trial/block without the need of continuously calculating the next trial/block, preventing any inter-trial/-block delay that could negatively impact a reaction time task like the IAT.

### Run Trial Procedure

The task was run block by block, and within each block we ran a trial procedure. First, the trial started with displaying the needed elements (e.g., the stimulus box) and hiding the unneeded elements (e.g., the fixation symbol). Second, it retrieved the stimulus from the pre-determined list and showed it promptly, and in the meantime, the timer started to generate a timestamp. Third, the program recorded the response, upon which another timestamp was created, such that we could calculate the reaction time for the trial using these two timestamps. After the response was recorded, the program advanced to the next trial automatically. As a side note, depending on the setting of the task, the IAT could stay at the same trial for a participant-initiated correction if an error response was entered.

### Save Data

For the customized question, we needed to use embedded data to save the IAT data. Specifically, we saved three sets of information for each trial: (1) the stimulus, (2) the correctness of the response, and (3) the reaction time. Because Qualtrics has a length limit for the embedded data fields, we saved these trial-related data by block to reduce the size. The embedded data became part of the final survey response.

## Discussion

In this article, we have described a mobile smoking research approach built on the Qualtrics survey platform and provided the technical guidance on the implementation of this approach for conducting mobile smoking research. This platform-agnostic approach does not require separate apps for specific mobile platforms (i.e., iOS and Android) and can even be completed on any device with internet access, including laptops, desktops, and tablets. This approach takes advantage of the versatility of Qualtrics as a survey tool, including its multiple question types, extensibility for question customization, unique survey links with desired expiration, and scalability with a wide range of APIs. Using a hypothetical study, we described how these components could be used to conduct mobile smoking research procedures, including screening, consenting, EMA, EMI, and behavioral assessment.

The approach described herein allows for a completely customizable platform that avoids the cost of project-specific app development, provided that researchers have access to the Qualtrics platform through their institutes' group license. While it does require considerable programming skills to put every element of the described approach into production, running smaller, more limited studies can be accomplished using only Qualtrics's built-in functionalities. All the procedures that require coding can be done in a non-programmatic manner, as all the involved components (e.g., SMS sending using AWS Pinpoint) have web interfaces designed for users who do not have the needed programming expertise.

However, if the research team does have programming expertise, the current report should provide sufficient technical details for them to deploy their mobile research using the described approach. Importantly, this approach is scalable because it can be used by multiple studies simply by including the study identifier as an additional field in the data tables and web service requests.

EMA has been an active paradigm employed by many tobacco researchers. The vehicle for administering EMA has evolved from handheld computers (33, 34) to smartphone-delivered apps (15, 17, 35). However, these platforms require the development of study-specific software that can be costly and time-consuming. Thus, to facilitate EMA-based research, it is necessary to develop alternative, affordable administration approaches. To this end, some researchers in the physical activity area were among the first to use SMS as prompts to conduct Qualtrics-based EMA research (36). In the present manuscript, we have extended their work by automating SMS scheduling and generating unique survey links for better data collection and tracking and described the pertinent technical implementation.

In the hypothetical study, the intervention mainly included NRT and phone-based counseling, consistent with the cessation guidelines. Besides these two treatment elements, to demonstrate how Qualtrics could be used for cessation treatment delivery, we included a third intervention element in the form of EMI: relapse prevention tailored to individual craving triggers, in the form of text messages (not SMS, a message displayed in the Qualtrics survey) or video clips. We understand that actual cessation mobile interventions may be more complicated and involve more interactive elements. Here, using a relatively simple form of mobile intervention based on participant-initiated ecological momentary assessments, our intention was just to demonstrate, as a proof-of-concept, that delivering personalized mobile interventions are possible with Qualtrics through the integration of custom JavaScript code to change HTML elements, offering an enormous possibility to researchers to develop any web-based mobile interventions.

Among the various aspects of programming to implement this approach, creating custom behavioral tasks in a Qualtrics survey requires special coding skills, and we understand that such requirement may constitute an obstacle to researchers who wish to integrate behavioral tasks into this approach. Thus, to facilitate survey-based IAT research, we have developed an open-source tool for preparing the needed custom JavaScript code (37). Looking forward, we are planning to expand the toolset to support other common behavioral tasks used in smoking research, allowing researchers to examine the possibility of using these behavioral tasks as novel intervention modalities for cessation treatment developments.

Another advantage of the current approach over smartphone apps is that researchers can conduct mobile smoking research projects in a platform-independent manner. If researchers wish to deploy an app-based mobile smoking intervention in a population, they need to make separate apps for different operation systems, mainly including Android and iOS. In contrast, the Qualtrics-based surveys use the smartphone's built-in web app, making all smartphones compatible with mobile smoking studies using Qualtrics as the data collection tool. However, it should be noted that relying on the web for data collection can be a shortcoming when a significant portion of participants may have internet access issues, a possible limiting factor that researchers must consider.

Although many researchers can take advantage of Qualtrics through their institutions' subscriptions, many do not have such subsidized access. Relatedly, another potential limiting factor is the scalability and generalizability of the Qualtrics-based research and treatment paradigms. Because of the reliance on Qualtrics, it is an open question how other researchers can easily implement the developed paradigm independent of the Qualtrics platform. Besides getting an individual or institutional Qualtrics subscription, tobacco researchers can consider launching a collaborative initiative to build a platform-agnostic, generalizable approach for conducting mobile smoking research.

In conclusion, the described Qualtrics-based approach is a cost-effective tool for conducting mobile smoking research. Although programming skills are required to deploy this approach in its full capacity to support multiple studies, researchers with insufficient programming skills can use the web interfaces of the involved elements if they are dealing with a small sample of participants.

## Data Availability Statement

The original contributions presented in the study are included in the article/supplementary material, further inquiries can be directed to the corresponding author.

## Author Contributions

YC completed the first draft of the manuscript. JR, RR, JM, and PC commented on the draft. RR led the technical development of the described approach. PC and JR obtained the funding for supporting the project. All authors contributed to the article and approved the submitted version.

## Funding

This project was supported by the U.S. National Institute on Drug Abuse Grant R01DA042526 awarded to PC and JR, by The University of Texas MD Anderson Cancer Center's Moonshot Program, and by MD Anderson's Cancer Center Support Grant (P30CA016672).

## Conflict of Interest

The authors declare that the research was conducted in the absence of any commercial or financial relationships that could be construed as a potential conflict of interest. The reviewer CS declared a past collaboration with one of the authors PC to the handling editor.

## Publisher's Note

All claims expressed in this article are solely those of the authors and do not necessarily represent those of their affiliated organizations, or those of the publisher, the editors and the reviewers. Any product that may be evaluated in this article, or claim that may be made by its manufacturer, is not guaranteed or endorsed by the publisher.
